# Conclusions from Two Model Concepts on Germinal Center Dynamics and Morphology

**DOI:** 10.1080/1044-6670310001597060

**Published:** 2002-12

**Authors:** Michael Meyer-Hermann, Tilo Beyer

**Affiliations:** Institute for Theoretical Physics Technical University Dresden Dresden D-01062 Germany

## Abstract

Germinal centers (GC) are an essential part of the humoral immune response. They develop a clear structure during maturation: Centroblasts and centrocytes are separated into two zones, the dark and the light zone. The mechanisms leading to this specific morphology as well as the reason for zone-depletion during a later phase of the GC reaction have not clearly been revealed in experiment. We discuss and weigh possible mechanisms of dark and light zone development in the framework of two mathematical models. In a comparative approach we formulate constraints on typical lymphocyte velocities in GCs which are characteristic for the different proposed mechanisms.


*Developmental Immunology*, December 2002 Vol. 9 (4), pp. 203–214

Taylor & Francis healthsciences

### Conclusions from Two Model Concepts on Germinal Center Dynamics and Morphology*

MICHAEL MEYER-HERMANN[†] and TILO BEYER

*Institute for Theoretical Physics, Technical University Dresden, D-01062 Dresden, Germany*

Germinal centers (GC) are an essential part of the humoral immune response. They develop a clear structure during maturation: Centroblasts and centrocytes are separated into two zones, the dark and the light zone. The mechanisms leading to this specific morphology as well as the reason for zone-depletion during a later phase of the GC reaction have not clearly been revealed in experiment. We discuss and weigh possible mechanisms of dark and light zone development in the framework of two mathematical models. In a comparative approach we formulate constraints on typical lymphocyte velocities in GCs which are characteristic for the different proposed mechanisms.

*Keywords*: Affinity maturation; B-cell kinetics; Dark and light zone; Germinal center; Immune system; Lymphocytes

### INTRODUCTION

An important part of the humoral immune response is the germinal center (GC) reaction. GCs are responsible for an optimization process of antibodies with respect to a specific antigen, the affinity maturation process: during the GC reaction new plasma cells are generated which secrete antibodies of considerably higher affinity to the antigen compared to the antibodies encoded by the originally activated B cells.

The GC reaction is initiated by antigen-activated B-cells that migrate into the follicle system. Here, they start to proliferate in the environment of follicular dendritic cells (FDCs). The initiation is believed to be of oligoclonal character, i.e. the number of seeder B-cells is small and of the order of three (Kroese *et al.*, 1987; Jacob *et al.*, 1991; Liu *et al.*, 1991; Küppers *et al.*, 1993). After three days of fast monoclonal expansion—the total number of proliferating B-cells (centroblasts) reaches about 12,000—a phase of somatic hypermutation is started (Jacob *et al.*, 1993; McHeyzer-Williams *et al.*, 1993; Pascual *et al.*, 1994; Han *et al.* 1995a). The diversity of encoded antibodies is enhanced in this way. The centroblasts differentiate into antibody-presenting centrocytes (Han *et al.*, 1997) and an apoptotic process is initiated. However, they have the possibility to get into interaction with the antigen-presenting FDCs and with T-helper cells. It is believed that this interaction depends on the affinity of antibody and antigen, and that those centrocytes which successfully bind the antigen are rescued from apoptosis (Liu *et al.*, 1989; Brandtzaeg, 1996; Tew *et al.*, 1997; Hollmann and Gerdes, 1999; Hur *et al.*, 2000; van Eijk *et al.*, 2001). This provides a more-step selection process (Lindhout *et al.*, 1997) of those B-cells with high affinity to the antigen. Positively selected B-cells further differentiate into plasma- and memory-cells (shortly denoted as output cells). In this way, the answer of the immune system is optimized with respect to the antigen.

The GC shows a very specific morphology. The proliferating and mutating centroblasts are collected in the dark zone. Centrocytes and FDCs build up the light zone. Such zones have been observed in experiments (Liu *et al.*, 1991; Camacho *et al.*, 1998). It has previously been shown that an intermediately appearing dark zone which is depleted in time is advantageous for affinity maturation (Meyer-Hermann, 2002a). The total duration of a GC reaction is about 21 days (Liu *et al.*, 1991; Jacob *et al.*, 1993; Kelsoe, 1996). Dark zones have been observed to appear at day 4 and to vanish at day 8 (Camacho *et al.*, 1998). However, there also exists evidence for dark zones of longer duration (Liu *et al.*, 1991).

In the present article we discuss and weigh possible mechanisms that may lead to the dark zone development. The analysis is based on two previously introduced models which will be denoted as the *signaling model* (Meyer-Hermann, 2002a) and the *chemotaxis model* (Beyer *et al.*, 2002), which are shortly recalled in the section Methods. The 2-dimensional (2D) signaling model is generalized to 3-dimensions (3D) and the compatibility of

*Presented at the Proceedings of the 4th Germinal Center Conference, June 2002, Groningen, The Netherlands.

[†]Corresponding author. Tel.: +49-351-463-33496. Fax: +49-351-463-37299. E-mail: meyer-hermann@physik.tu-dresden.de

ISSN 1044-6672 print © 2002 Taylor & Francis Ltd
DOI: 10.1080/1044-6670310001597060

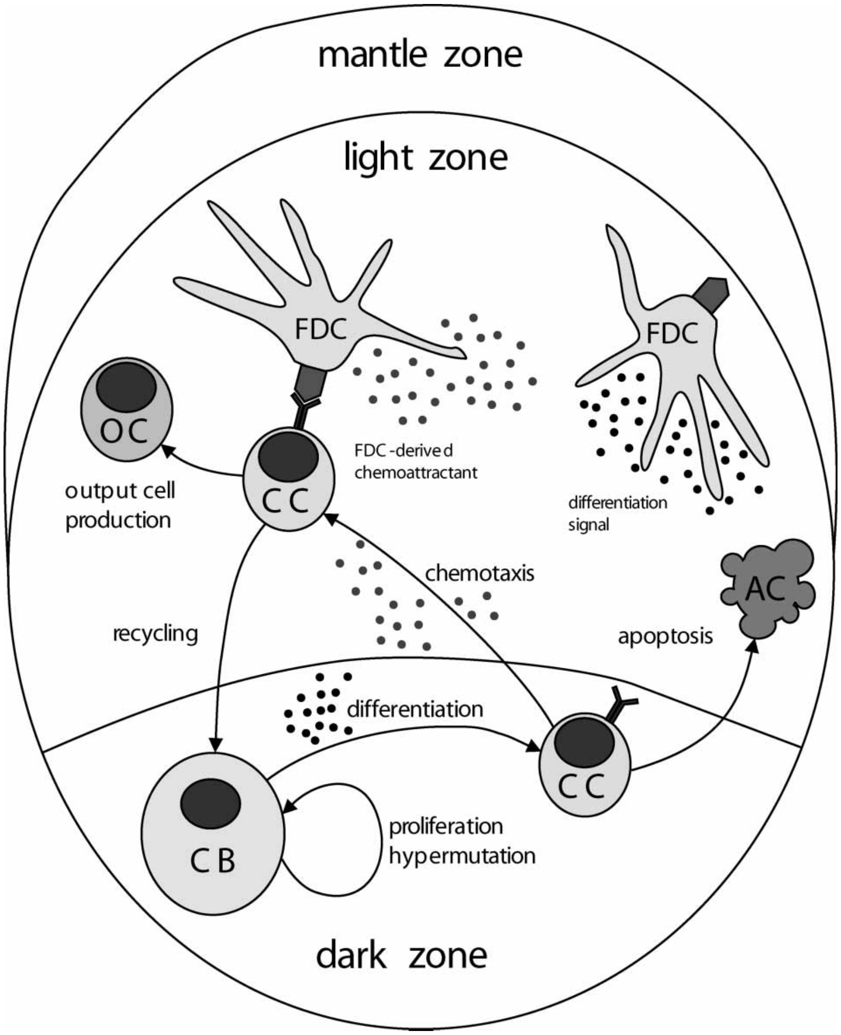

FIGURE 1 A schematic view on the GC reaction: the differentiation and interaction processes included in both, the signaling and the chemotaxis model are shown. In addition, a differentiation signal and a chemotactic signal are investigated.

the results in 2D and 3D is considered in the section Results. In a second step, the 3D results are compared to those gained with the 3D chemotaxis model. The concepts of both models are combined and new possible pathways for dark zone development are deduced. The results are evaluated in the section Discussion, where some pathways are excluded and others favored. Especially, we will discuss the relevance of cell velocities in GCs as well as constraints from cell population kinetics. The above models are up to now the only models allowing investigation of the origin of dark zones as other mathematical models of the GC do not include spatial aspects explicitly (Oprea and Perelson, 1996, 1997; Rundell *et al.*, 1998; Kesmir and de Boer, 1999; Oprea *et al.*, 2000; Meyer-Hermann *et al.*, 2001, 2002b).

### METHODS

A short description of two previously introduced mathematical models for the morphological organization and cell dynamics of the GC (Meyer-Hermann, 2002a; Beyer *et al.*, 2002) is provided in this section. In both models, the GC is simulated with a cellular automation based approach on an equidistant lattice. Both models include (see Fig. 1)

- centroblast proliferation,
- somatic hypermutation of centroblasts,
- centroblast differentiation to centrocytes,
- centrocyte apoptosis,
- centrocyte-FDC interaction depending on the anti-body-antigen affinity,
- positive centrocyte selection (inhibition of apoptosis),
- centrocyte recycling to a re-proliferating stage, and
- centrocyte differentiation to plasma- and memory cells.

The affinity of the encoded antibodies to the antigen is formulated with the well known shape space concept (Perelson and Oster, 1979). Each type of antibody is represented on a four-dimensional lattice which is ordered in such a way, that the affinity to the antigen is quasi continuously changed between neighboring points.

Somatic hypermutation is represented by a jump to a neighbor point. The affinity between antibodies on centrocytes and antigens held on FDCs is modeled by a Gaussian affinity weight function centered at the optimal antibody type (Meyer-Hermann *et al.*, 2001).

Both models divide the GC reaction into three phases: A phase of monoclonal expansion of centroblasts (3 days), a phase of primary optimization, already including all above mentioned processes with exception of differentiation into plasma- and memory cells (2 days) (see Meyer-Hermann, 2002a), and a phase of output production lasting until the end of the reaction which differs from the previous phase by the onset of differentiation to plasma- and memory cells.

The models differ in some spatial aspects corresponding to different subjects we focused. The *signaling model* (Meyer-Hermann, 2002a,b) concentrates on non-local interactions between FDCs and centroblasts using a signal molecule which initiates differentiation processes. The *chemotaxis model* (Beyer *et al.*, 2002) focuses on the aspect of cell movement, where a random movement is superposed to a movement induced by a chemotactic signal. In the following, the specific properties of both models are recalled.

### The Signaling Model

The GC is simulated on an equidistant cubic lattice with lattice constant 10 μm. This corresponds to the average cell diameter of B-cells in GCs. The radius of the lattice varies between 160 and 220 μm, corresponding to typical radii of GCs. Each lattice point can be occupied by exactly one centroblast, centrocyte, or output cell. All cells move on the lattice in an undirected and random way. The diffusion constants are adapted corresponding to the different diameters of centroblasts and centrocytes (Kroese *et al.*, 1987; Liu *et al.*, 1994; Hostager *et al.*, 2000), in order to guarantee a cell motility that corresponds to the cell volume. FDCs are represented by an immobile soma at one lattice point and four (in 2 dimensions) dendritic arms of 30 μm length.

It has previously been shown that the development of dark zones requires a non-local cell–cell interaction (Meyer-Hermann, 2002a). The signaling model introduces a diffusing signal molecule, which is produced by FDCs or T-cells and bound by centroblasts. Note that this implies a separation of signals acting on proliferation and differentiation of centroblasts, as has been proposed in corresponding experiments (Han *et al.*, 1995b). The signal molecules diffuse on the lattice according to a classical diffusion equation. The diffusion is not influenced by the presence of cells at the same lattice point. The molecules are represented in quanta which are assumed to contain enough signal molecules necessary to initiate the centroblast differentiation process to centrocytes. Using this non-local concept in a 2D model an intermediately appearing dark zone occurs. The duration of the dark zone basically depends on the amount of secreted signal molecules and its diffusion constant. The ratio of centroblast differentiation and proliferation rates changes the duration of the dark zone as well. However, this ratio has influence also on the total life time of the GC as a whole and, therefore, is determined independently. For more details concerning the signaling model we refer to (Meyer-Hermann, 2002a).

### The Chemotaxis Model

The description of a chemotactic response of centroblasts or centrocytes to a corresponding signal makes necessary a more detailed spatial prescription. The cell velocities attained are substantially higher compared to the purely undirected cell movement in the signaling model. The model is based on a regular face centered cubic lattice. The major pillar of the chemotactic model is a consistent space description (Beyer *et al.*, 2002). This includes the following model properties.

- The volume occupied by the cells at one lattice point (and for large cells as for centroblasts also on the neighboring lattice points) is calculated explicitly according to the actual radius of the cell.
- The cells grow according to cell-eigentime and to the space available in the direct environment. This applies to the growth process of recycled and non-recycled centroblasts.
- The cells shrink according to a dynamical equation. This applies to the differentiation process of centroblasts to centrocytes during which the cell volume is reduced to approximately 1/12 of the centroblast volume.
- Centroblast proliferate in a volume conserving way. This implies two cells being present at one lattice point for a certain while. The cells then tend to find free neighbor points in order to acquire space for cell growth.
- The movement of cells is a superposition of an undirected random movement (as in the signaling model) and a directed movement directed by the chemotaxis field.
- The centroblast differentiation process to centrocytes is governed by a diffusing signal molecule which is assumed to be homogeneously distributed over the GC volume. This differs from the signaling model. However, diffusing signal molecules will also be considered in the chemotaxis model (see section "On the origin of GC dark zones").

The source of the chemotactic field has not been specified until now. Indeed, this has been a major parameter of our model. We considered the FDCs or cells in the mantle zone as source of a chemotactic signal. We found that a superposition of FDC- and mantle zone-derived chemotactic signals can account for sorting of centroblasts and centrocytes. The shape of the thus induced dark zone appeared to be unphysiological.

For more details concerning the chemotaxis model, we refer to Beyer *et al.* (2002).

### Initialization of Simulations

The simulations are started with three randomly distributed seeder B-cells and a constant FDC density that provides enough interaction points for centrocytes. The fact that centroblasts proliferate at least in parts outside the FDC network turned out to be a necessary requirement for the development of dark zones (Meyer-Hermann, 2002a). This is ensured by a random distribution of the FDCs on one hemisphere of the total GC volume that is achieved at the peak of the reaction. The seeder cells are of low but non-vanishing affinity to the antigen. They can reach the optimal antibody-type with 5–10 mutations (Küppers *et al.*, 1993; Wedemayer *et al.*, 1997). The simulations are insensitive to a change of the time-step-width which ranges between 0.01 and 0.1 h for the presented results. In contrast to 2D simulations where the results depend on the used generator of random numbers and the stability of the results becomes small for small cell numbers (which especially is important at the end of the reaction), the 3D simulations produce rather stable results.

### RESULTS

The presentation of the results is divided into two parts. At first, we will compare simulations of GC reaction based on the *signaling model* in two and three dimensions. Thus having established a three-dimensional model for the GC reaction, the simulation results will be compared to results generated with the *chemotaxis model*. The main focus lies on the analysis of necessary and sufficient conditions for the development of dark and light zones in GC reactions. This includes requirements for the separation of centroblasts and centrocytes, the shape of resulting dark zones, as well as aspects of GC volume kinetics and affinity maturation.

### A Comparison of 2D and 3D Simulations

The basic question in this section is whether the 2-dimensional (2D) simulation results presented in Meyer-Hermann (2002a) may be considered as representative for 3-dimensional (3D) simulations. This is necessary in order to compare the simulation results to real GC reactions which naturally are 3D. Especially the parameter values used in the 2D simulation are concerned. How do they change during the transition from 2D to 3D simulations? If the simulations in both dimensions are consistent, the parameter values in the 3D model should follow from the 2D model parameters in a straight forward way. Basically, the rates for proliferation, differentiation, etc. have to be multiplied with 3/2 in order to guarantee comparable simulation results in 2D and 3D.

Note, that this transition rule is an inversion of the procedure pursued in the 2D model (Meyer-Hermann, 2002a). Here, physiological constraints have been established for the parameter values from experiment and the values have been incorporated into the model with an additional factor of 2/3. For example, the proliferation rate of centroblasts is known to be 1/6 h while 1/9 h has been used in the 2D model. In fact this procedure already assumes a correct representation of 3D GC by the 2D model. Therefore, the present analysis may be interpreted as an *a posteriori* justification of the 2D model results.

The maximum volume of the GC reaction is chosen such that the number of centroblasts and centrocytes together does not exceed 13,000, which is the total cell number expected from experiment in fully developed GCs. The number of FDCs (now having 6 instead of 4 dendritic arms) is accordingly corrected in order to guarantee an unchanged FDC density in the GC. This corresponds with respect to the relative number of interaction points for centrocytes and the density of differentiation signal sources (compared to 2D simulations). We expect that due to the larger total number of lymphocytes, affinity maturation will be slightly optimized with respect to 2D simulations. Once having higher affinity to the antigen in average, the probability of positive selection of centrocytes will be enhanced, possibly implying a non-declining cell population in the late phase of the GC reaction. This indeed is the case and may be accounted for by a slightly higher centroblast differentiation rate. This parameter (in relation to the proliferation rate fixed by experiment) determines the late-phase behavior of the GC reaction. However, to illustrate the direct correspondence of the 2D and 3D simulations, all rates are modified by the above mentioned factor of 3/2 resulting in the parameter values given in Table I ((s) and (s1)). A section through the simulated GC is shown in Fig. 2, where the section is chosen in correspondence to the polarity defined by the FDC distribution.

The intermediately appearing dark zone is found in complete similarity to the 2D simulation results (see Meyer-Hermann, 2002a, Fig. 3). In an early phase, the signal molecules act at the border of the dark zone only and induce centroblast differentiation at the dark zone surface pointing towards the FDC network. The differentiation signal needs some time to penetrate the dark zone and to dissolve it (see Fig. 3). In order to get an intermediate dark zone, the signal production rate has to overcome some critical value. Otherwise the whole GC reaction is exploding because the process of proliferation dominates the differentiation process and centroblasts accumulate. One may speculate that a subcritical differentiation signal production may lead to GC having the morphology of malignant GCs (Hollowood and Macartney, 1992; Zelenetz *et al.*, 1992; Brauninger *et al.*, 1999; Küppers, 1999; Marafioti *et al.*, 1999).

The general kinetics are found to be very similar as well (see Fig. 4). As before, the population grows exponentially. The growth is slowed down when

TABLE I The model parameter values: the parameter values as used in 2D (s) and 3D (s1) simulations using the signaling model are shown in the two left columns. The rates in 3D compare to the values in 2D simulations by an additional factor of 1.5. The right columns show the parameter values as used to compare the 3D signaling model (s2) with the chemotaxis model (c).

| Parameter | 2D (s) | 3D (s1) | 3D (s2) | 3D (c) |
|---|---|---|---|---|
| Shape space dimension | 4 | 4 | 4 | 4 |
| Width of Gaussian affinity weight function | 2.8 | 2.8 | 2.8 | 2.8 |
| Lattice constant | 10 μm | 10 μm | 10 μm | 10.6 μm |
| Radius of GC | 220 μm | 160 μm | 160 μm | 172.5 μm |
| Number of seeder cells | 3 | 3 | 3 | 3 |
| Diffusion constant for centroblasts | $5\,\frac{\mu m^2}{h}$ | $5\,\frac{\mu m^2}{h}$ | $5\,\frac{\mu m^2}{h}$ | $5\,\frac{\mu m^2}{h}$ |
| Ratio of centroblast to centrocyte radius | 3 | 3 | 2.3 | 2.3 |
| Diffusion constant of signal molecules | $200\,\frac{\mu m^2}{h}$ | $200\,\frac{\mu m^2}{h}$ | $200\,\frac{\mu m^2}{h}$ | $200\,\frac{\mu m^2}{h}$ |
| Number of FDCs | 20 | 225 | 172 | 215 |
| Length of FDC arms | 30 μm | 30 μm | 30 μm | 42 μm |
| Duration of phase of monoclonal expansion | 72 h | 72 h | 72 h | 72 h |
| Duration of optimization phase | 48 h | 48 h | 48 h | 48 h |
| Rate of proliferation (2D) | 1/9 h | 1/6 h | 1/6 h | 1/6 h |
| Maximal distance for CB proliferation | 60 μm | 60 μm | 60 μm | – |
| Mutation probability | 0.5 | 0.5 | 0.5 | 0.5 |
| Signal production rate by FDCs | 9/h | 15/h | 27/h | 13/h |
| Rate of centroblast differentiation | 1/6 h | 1/4 h | 1/3 h | 1/3 h |
| Rate of FDC-centrocyte dissociation | 1/2 h | 1/1.3 h | 1/3 h | 1/3 h |
| Differentiation rate of selected centrocyte | 1/7 h | 1/4.7 h | 1/3.5 h | – |
| Recycling probability of selected centrocyte | 0.8 | 0.8 | 0.8 | 0.8 |
| Rate of centrocyte apoptosis | 1/7 h | 1/4.7 h | 1/6 h | 1/6 h |
| Centrocyte chemotaxis | – | – | – | $13\,\frac{\mu m}{min}$ |
| Centroblast chemotaxis | – | – | – | $0.1\,\frac{\mu m}{min}$ |

centroblast differentiation starts and the population reaches a maximum after about 4 days. Note that the maximum is slightly retarded for 2D which is related to the statistical properties of both simulations. Statistical fluctuations in 2D simulation results are strongly reduced in 3D. This simply goes back to the overall higher number of cells in the GC, which differs by more than an order of magnitude. Correspondingly, the curves exhibit higher stability and less noise fluctuations. However, in the very beginning of the simulation the number of seeder cells is still 3 (i.e. a small cell number) and implies a slight dependence of the simulation on the early (random) proliferation behavior of centroblasts. This finds expression in a slight shift of the lymphocyte peak number in time. The retardation is then transported through the later phases of the GC reaction.

The still low affinity of the lymphocytes after 4 days implies a high probability of apoptosis resulting in a nearly exponentially declining lymphocyte population. When the average affinity of the cells becomes higher, the reduction of cell population becomes nearly linear (thus becomes slower) and reaches a plateau population with comparably small cell numbers. How this low level GC reaction can be terminated remains a problem. Note that this problem is even more pronounced for 3D as here the total lymphocyte population seems to grow at the very end of the reaction. This clearly points towards an additional mechanism that stops the reaction (see section "Discussion").

The affinity maturation process is compared in Fig. 5. It seems as if the fraction of high affinity centroblasts and centrocytes is larger in 2D compared to 3D in the final phase of the GC reaction. However, the difference lies within statistical fluctuations of 2D results. The number of lymphocytes in the late phase of 2D simulations is extremely small and therefore, statistical uncertainties become relevant.

As in the 2D simulation, the four phases of affinity maturation are confirmed in an even more pronounced way in 3D: during monoclonal expansion, no high affinity cells may appear. When somatic hypermutation is started the fraction of high affinity cells moderately increases—what we denote by the *primary optimization phase*. It follows a phase of steep increase which is characterized by the depletion of the dark zone containing unrecycled centroblasts of below average affinity to the antigen. In this sense, the depletion of the dark zone turns out to be essential for a successful GC reaction. During this *secondary optimization phase*, a fine tuning of already positively selected, i.e. recycled lymphocytes is put forward. Finally, the affinity maturation process saturates on a high level. The exact level has to stay below 1 as even optimal lymphocytes, when recycled, continue somatic hypermutation and therefore may also reduce their affinity to the antigen. The saturation level is a result of a mutation flow of cells into and out of the optimal clone.

The total (time integrated) production of plasma- and memory cells (also shown in Fig. 5) is of higher affinity to the antigen in 3D than in 2D. This corresponds to the expected implications from the substantially larger pool of mutating centroblasts. Already in an early phase the probability of finding optimal clones is considerably enhanced in 3D due to a larger lymphocyte diversity. As a consequence, the output quality starts on a higher level producing a difference which is not equilibrated during the whole GC reaction. This shift towards higher affinities

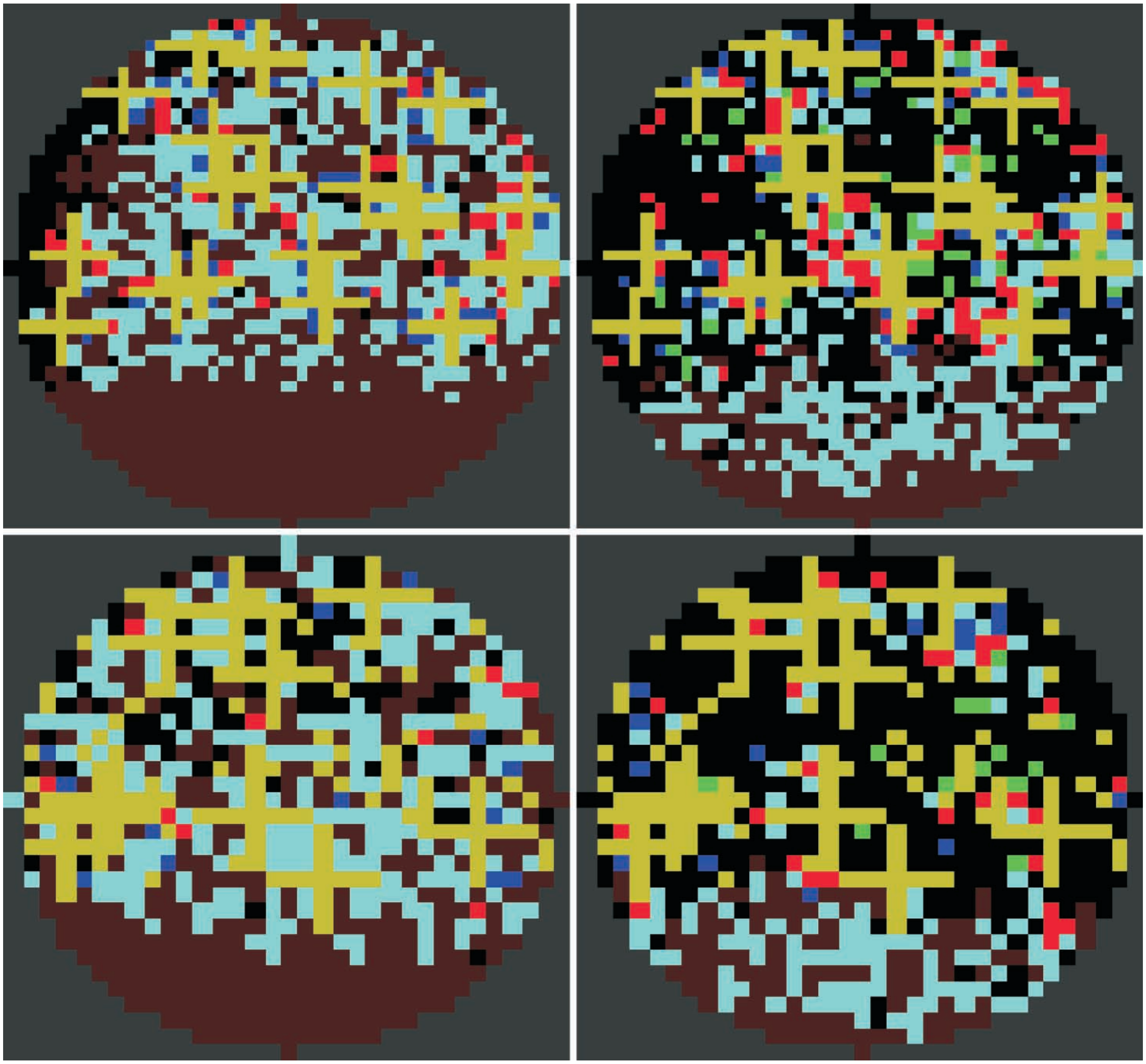

FIGURE 2 Sections through the signaling model GC: a section through the 2D (upper row) and 3D (lower row) simulated GC reaction is shown at days 4 and 8. Centroblasts (dark red) build a dark zone besides the FDC (yellow) network. Centrocytes (cyan, blue) are positively selected (blue) in interaction with FDCs. They further differentiate to plasma or memory cells (green) or recycle back into a re-proliferating cell state, i.e. to recycled centroblasts (light red). The dark zone already present at day 4 is depleted around day 9.

of output cells is found to be systematic throughout all simulation runs.

### On the Origin of GC Dark Zones

Previously, two possible origins of GC dark zones have been investigated. In the framework of the *signaling model*, it turned out that dark zones develop provided that the centroblasts proliferate at least in parts beyond the FDC network and that a (yet unknown) signal molecule is secreted by FDCs, diffuses over the GC, is bound by centroblasts, and initiates centroblast differentiation to centrocytes (Meyer-Hermann, 2002a). The essential point is the non-locality of the interaction between FDCs and centroblasts. Local interactions could be excluded as a relevant process driving GC zoning. From the above analysis, we can conclude these results to remain valid in 3D simulations.

Within the framework of the *chemotaxis model* incorporating a more sophisticated volume and cell motility concept we found that chemotaxis alone (again providing a non-local interaction with lymphocytes) may separate centroblasts and centrocytes from each other. The resulting dark zones have non-physiological shapes at least for human and mice GCs (Beyer *et al.*, 2002). However, such ring shaped dark zones are found in chicken (Yasuda *et al.*, 1998).

In the following we will combine both concepts and focus on two basic questions: does the more detailed volume and cell motility concept used in the chemotaxis

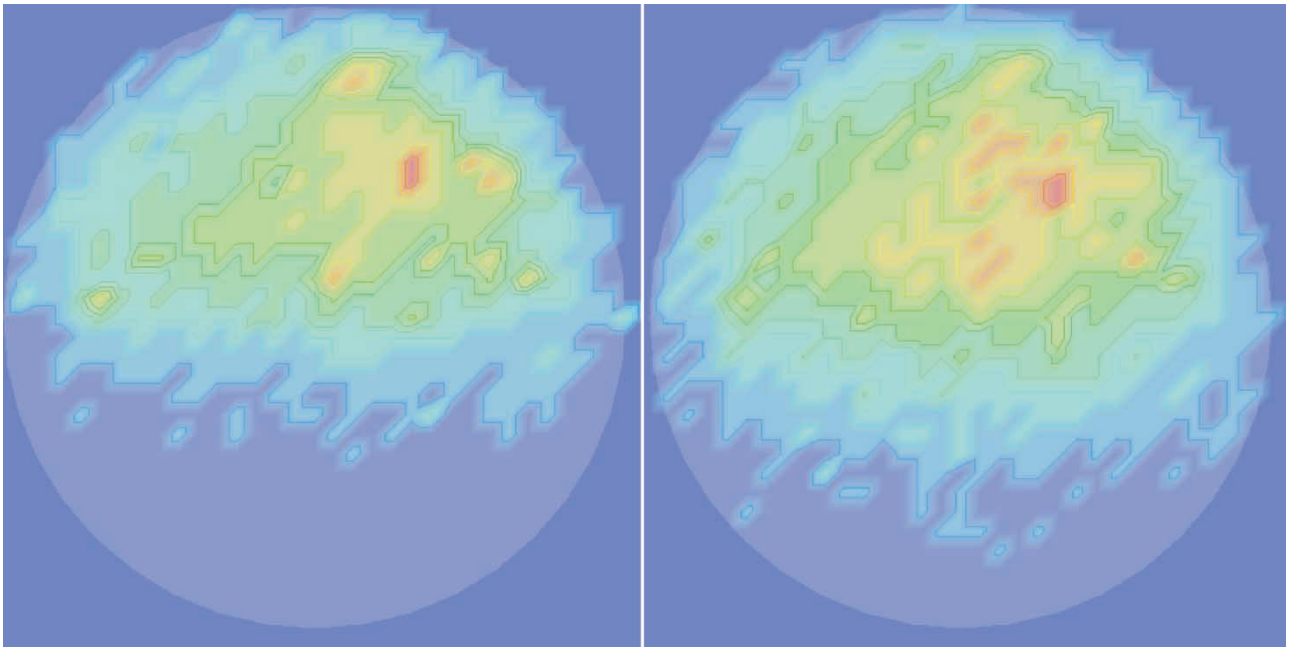

FIGURE 3 Differentiation signal concentration: the signal concentration is shown on the same section as in Fig. 2 (3D). At day 4 the differentiation signal has still low concentration and is limited in diffusion by the signal consuming centroblasts in the dark zone. At day 8 the differentiation already penetrates the dark zone and starts to destroy it by inducing centroblast differentiation. The signal concentration increases from blue to red.

model alter the results found in the signaling model? In other words, is cell motility appropriately described in the signaling model? And, does a differentiation signal provide a possibility to optimize the shape of dark zones that develop due to chemotactic signals?

To this end we introduce into the chemotaxis model a FDC-derived differentiation signal diffusing over the GC volume and inducing centroblast differentiation to centrocytes. The model parameters are summarized in Table I (c). The signaling model is modified correspondingly in order to become comparable to the chemotaxis model: the FDC network is reduced to 50% of the maximum GC volume. The dendritic arms of the FDCs are considered to be transparent in the sense that lymphocytes are allowed to be at the same position on the lattice as the dendrites. This remains forbidden for the FDC soma. Such a modification should have an impact on the motility of the cells as the FDC network effectively becomes less dense.

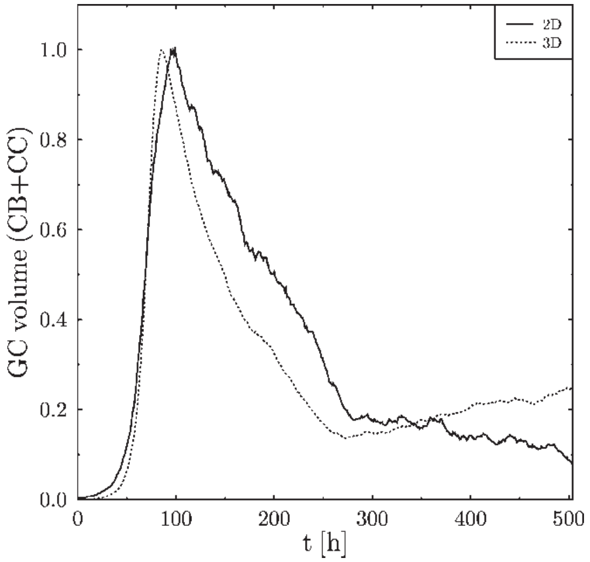

FIGURE 4 The time course of the GC volume: in order to compare both GC volume kinetics, each time course has been normalized to its maximum value. The 2D simulation reaches its maximum slightly later compared to the 3D simulation. This retardation is transported into the later phases of the GC reaction. The cell population declines less in the 3D simulation during the final phase of the GC reaction.

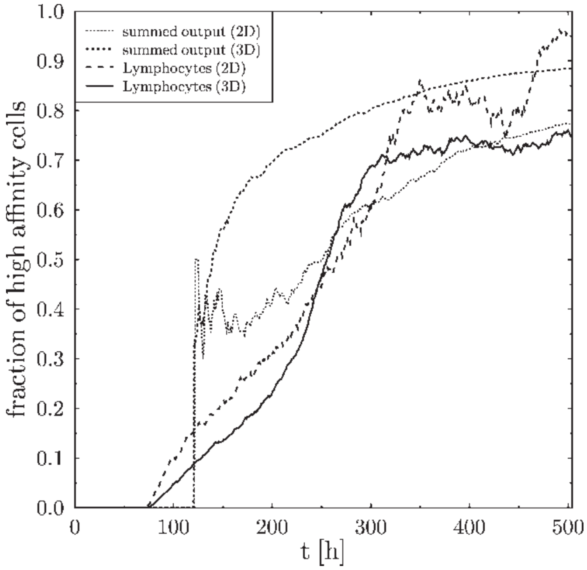

FIGURE 5 Affinity maturation: the four phases of the affinity maturation process are even more clearly seen in 3D simulations compared to 2D. Note that the summed total production of plasma and memory cells is of substantially higher quality in 3D than in 2D. Statistical fluctuations seen in 2D simulations are smoothened in 3D.

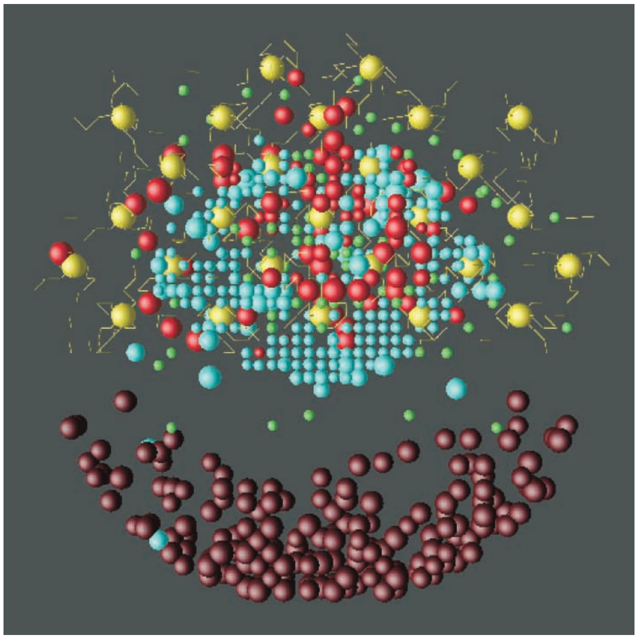

FIGURE 6 A gap between dark and light zone: a GC simulation (day 8) using the chemotaxis model, with a FDC-derived chemotactic signal acting on centrocytes, and with a centroblast differentiation signal diffusing on the GC volume. In this scenario, an unphysiological gap develops between dark and light zones. Centroblasts (dark red), recycled centroblasts (light red), centrocytes (cyan), output cells (green), FDCs (yellow).

The number of FDCs is chosen such that a constant FDC density is ensured inside the FDC network in both models. All the modifications with respect to the 2D signaling model are summarized in Table I (s2). Note that besides a correction of the differentiation signal production (due to slightly different GC volumes) the parameters are identical in both models. We also used the same type of seeder cells with starting affinity to the antigen of 0.04.

At first, we checked that the chemotaxis model indeed reproduces the results of the signaling model. These are simulated by switching off the chemotaxis response of all lymphocytes which should project the chemotaxis model on the signaling model. Compared to the signaling model, the results indeed remain unchanged concerning the general GC volume kinetics, affinity maturation, as well as the intermediately appearing dark zone (data not shown).

In a second step, we combined the diffusing differentiation signal with a chemotactic response of centrocytes. As already found for a homogeneously distributed differentiation signal, a dark zone develops because all centrocytes emerging through centroblast differentiation within this area are driven towards the FDC network. The dark zone appears to be sickle shaped and a gap between the proliferating centroblasts and the FDC network develops (see Fig. 6). This does not happen in the signaling model because of the lack of an attractant for centrocytes in the FDC network. Here, centrocytes find their interaction partner by an undirected movement thus filling the gap between proliferating dark zone and FDC network. In fact, the main part of the affinity maturation process is done in the borderland of dark and light zone in the signaling model which appears more realistic.

In order to avoid the development of such a gap we let the centroblasts weakly respond to the chemotactic signal as well. Indeed, the gap vanishes in this scenario. Note, that the gap may also vanish by changing the boundary conditions or by introducing cell adhesion into the model. Corresponding cell adhesion molecules allowing for an interaction of lymphocytes and FDCs have indeed been identified in experiment (see Koopman *et al.*, 1991).

The development of the GC reaction under these assumptions is shown in Fig. 7 and compared to the corresponding result of the signaling model. While in the enlarged chemotaxis model, the dark and light zone nicely develop, the signaling model shows a well developed dark zone only. The light zone is poorly populated at day 8 and most centrocytes remain at the border of the FDC network. This is a clearly unphysiological result which will be further discussed in section "Discussion".

The kinetics of the total GC cell population as found in the signaling and chemotaxis model are compared in Fig. 8. After a nearly exponential cell population growth, it reaches a maximum after about 4 days, then rapidly declines, reaches a more linearly decreasing phase, and finally stabilizes on a low level. Even showing a rather similar shape in the main part of the GC reaction, there is a crucial difference between both models in the final phase. While the cell population steadily declines in the chemotaxis model, this is not the case in the signaling model. We discussed the necessity of an additional mechanism that stops the GC reaction in the very late phase of the reaction (Meyer-Hermann, 2002a). This is confirmed in this analysis for the signaling model. It is important to realize that such an additional mechanism is not necessary to explain the main course

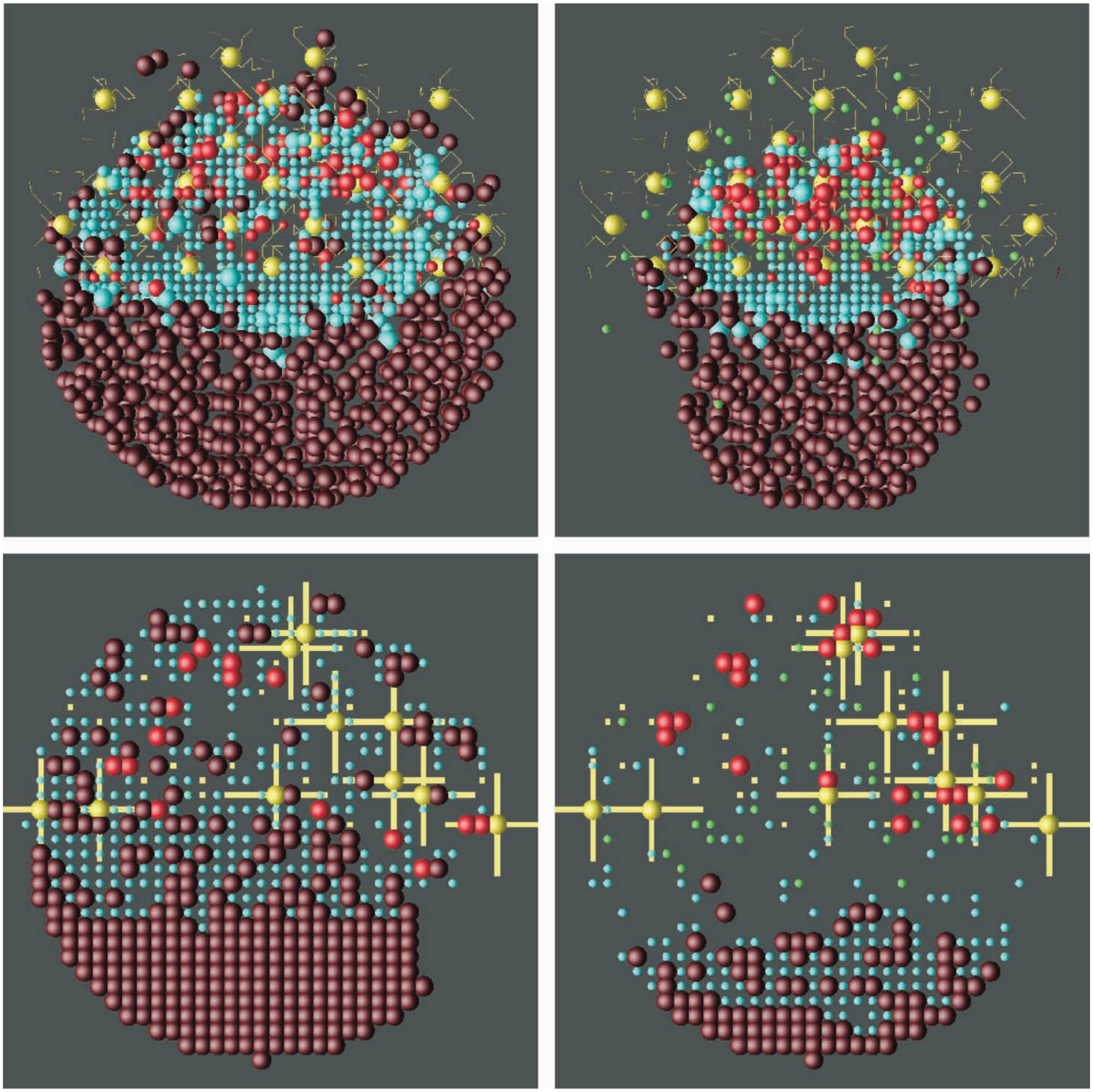

FIGURE 7 Comparing sections through GCs in the signaling and the chemotaxis model: a GC simulation (day 4 and 8, upper row) using the chemotaxis model, with a FDC-derived chemotactic signal acting on centrocytes (blue) and weakly on centroblasts (dark red), and with a centroblast differentiation signal diffusing on the GC volume (upper row). The unphysiological gap (see Fig. 6) has vanished. These sections compare to the ones generated with the signaling model (lower row) based on the same parameter set (see Table I, (s2)). Recycled centroblasts (light red) are found in the light zone; plasma- and memory cells (green); FDCs (yellow).

of the cell population dynamics which stays in agreement with experiment (Liu *et al.*, 1991; Hollowood and Macartney, 1992; Meyer-Hermann, 2002a, Fig. 12).

We had to impose different conditions on the undirected cell motility (not on the directed movement induced by chemotaxis). Originally, the lymphocytes were considerably faster in the chemotaxis model compared to the signaling model. We could even determine a lower limit for the undirected cell velocity in order to achieve cell sorting in GCs due to a chemotactic attractant (Beyer *et al.*, 2002). In this model, the centroblast differentiation signal was homogeneously distributed on the total GC reaction volume, i.e. it might be considered to diffuse considerably faster compared to the typical time scale of the GC reaction. Now, combining chemotaxis with a slowly diffusing signal molecule, thus generating an inhomogeneous distribution of differentiation signal concentration (compare Fig. 3), the (undirected) cell motility has to be reduced down to the level of typical cell velocities as used in the signaling model. For larger cell velocities, the dark zone becomes ring shaped as it does for a homogeneously distributed differentiation signal

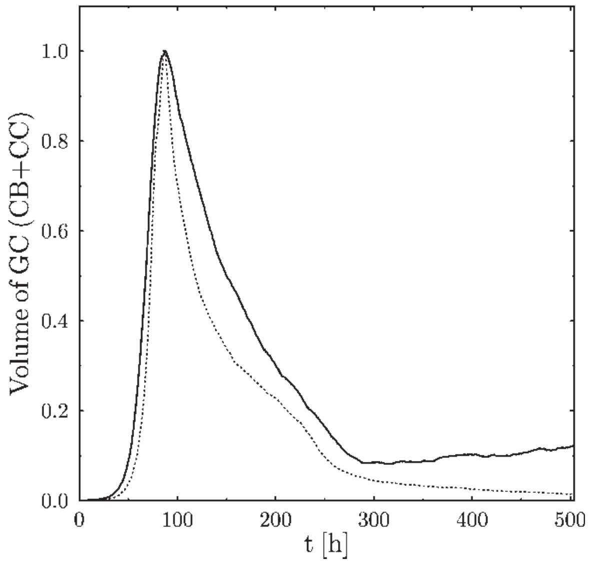

FIGURE 8 Comparing the time course of the GC volume: both time course has been normalized to its maximum value. The cell population kinetics is found to be similar in the signaling (full line) and the chemotaxis model (dotted line). Note the end phase of the GC reaction which seems not to stop in simulations with the signaling model.

(see Beyer *et al.*, 2002, Fig. 2,6). This implies that the ring shape of the dark zone found in the chemotaxis model is related to high cell velocities compared to the signaling model. The shape of the dark zones generated in the chemotaxis model is optimized with respect to physiological shapes by reducing typical cell velocities and by using diffusive differentiation signals.

Finally, we compare the affinity maturation process in both models in Fig. 9. The time course of lymphocyte affinity to the antigen is very similar in both models.

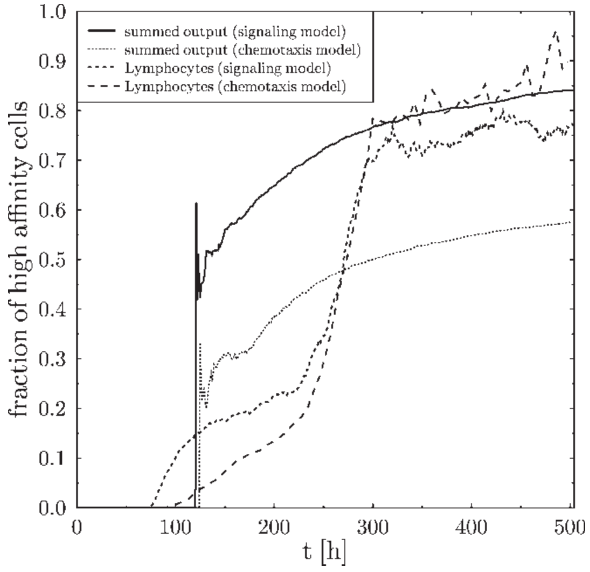

FIGURE 9 Comparing affinity maturation: the fraction of high affinity cells (affinity is larger than 0.30) is very similar for lymphocytes in simulations generated with the signaling and chemotaxis model, respectively. However, the (time integrated) total number of produced plasma- and memory cells differs considerably.

The already discussed four phases of the affinity maturation process are clearly seen. However, there is a large difference for the total (time integrated) output affinity, which is substantially higher in the signaling model. The higher output quality mirrors the slightly higher fraction of high affinity cells between days 5–8, i.e. immediately after the onset of output production. At this time point the total number of cells is large, and therefore, slight differences in average affinity have a huge impact on the time integrated output quality. This interpretation in confirmed by the fact that after the initial peak, the output quality develops similarly in both models, i.e. the curves are basically parallel to each other in Fig. 9. The initial head start of the signaling model is transported up to the end of the reaction.

### DISCUSSION

We introduced a 3 dimensional generalization of a previously introduced 2 dimensional model for the GC reaction. The results found in 2D before are basically confirmed in 3D simulations when the parameter values are adopted in a straight forward procedure:

- An inhomogeneous FDC network and a non-local interaction between FDCs and centroblasts (realized by a centroblast differentiation signal) are a sufficient condition for the development of dark zones. Such dark zones appear intermediately and vanish around day 9 of the GC reaction.
- The cell population kinetics are very similar in the main part of the GC reaction. However, the already suspected problem of a signal that stops the reaction reappeared in a more important manner in 3D simulations.
- Affinity maturation shows the same four phases as in 2D: monoclonal expansion, primary optimization (with somatic hypermutation), fine tuning of recycled lymphocytes and depletion of the dark zone, and finally a saturation in affinity. Affinity maturation works better in 3D as the diversity of lymphocytes is enhanced in 3D due to higher cell numbers. Therefore, optimal clones are found earlier in the course of the GC reaction and the total output quality is higher.

The satisfying correspondence of the 2D and 3D version of the signaling model *a posteriori* justifies the interpretation of the 2D results (Meyer-Hermann, 2002a).

The now tested 3D signaling model has been compared to the results of the 3D chemotaxis model. The analysis showed that the self-consistent volume concept used in the chemotaxis model is not primarily important when considering slowly moving lymphocytes in the framework of the signaling model. The restriction on a more naive motility concept does not affect the results.

It has previously been found that an FDC-derived chemotactic signal acting on centrocytes is able to separate centroblasts and centrocytes, thus generating a dark and a light zone. However, the shape of the dark zone becomes ring or sickle shaped–in contradiction to experiment. In this investigation, the differentiation signal was homogeneously distributed on the GC volume. Here, we combined the chemotaxis model with a diffusing differentiation signal as used in the signaling model. In addition, we assumed centroblasts to respond to the chemotactic signal as well but about 2 orders of magnitude weaker. This avoids an unphysiological gap between dark and light zone. The interesting result is that in order to get a reasonable shape of the dark zone, the undirected (random) cell velocity has to be reduced to values that are comparable to the ones that have been used in the signaling model before.

This implies two possible scenarios concerning typical cell velocities in GC reactions:

1. If lymphocytes are slowly moving in GCs (with about 1 μm/h) the concept of a slowly diffusing signal becomes rather attractive. Chemotaxis may be existent in this case but primarily has the role to cluster the lymphocytes in the GC.
2. If lymphocytes are rapidly moving in GCs (with about 100 μm/h), the signaling model is ruled out as mechanism for GC zoning. This does not necessarily imply that chemotaxis is the major player. We recall, that the shape of dark zones induced by chemotaxis alone is unphysiological—a fact being related to high cell velocities. There has to exist another supplementary process which generates the correct shape.

Thus, typical cell velocities if measured would distinguish which of these scenarios is realized in nature.

The fact that the light zone is poorly populated in the signaling model (see Fig. 7) is, in part, an artefact of the model principles. The simplified volume concept implies that in a centrocyte dominated area, the space is basically empty for the diameter of centrocytes is considerably smaller compared to the lattice constant. However, the problem goes beyond this explanation and a careful analysis reveals to conflicting tendencies. In order to ensure a declining cell population in the late phase of the GC reaction, the centroblast differentiation rate has to be large enough (Meyer-Hermann *et al.*, 2001). A large differentiation rate reduces the chance of positively selected centrocytes (typically staying in the light zone) to re-proliferate after recycling. It is exactly this process that ensures a dense population in the light zone. Indeed, smaller differentiation rates repopulate the light zone but let the GC cell population explode in the late phase. Consequently, again we are led to two alternative pathways:

1. There exists an additional mechanism stopping the GC reaction. Such a mechanism appeared to be necessary already before (see Figs. 4 and 8 as well as Meyer-Hermann (2002a)). In this case the centroblast differentiation rate could be smaller during the highly populated phase of the GC reaction and the light zone would become densely populated.
2. The centrocytes are driven towards the FDC network by a chemotactic signal. This exactly corresponds to the enlarged version of the chemotaxis model (combined with a diffusing differentiation signal) which has been presented here (see Fig. 7).

From two different angles we are led to the conclusion that neither diffusing differentiation signals nor chemotaxis alone can account for both a physiologically shaped dark zone and correct cell population dynamics. Chemotaxis fails to explain the dark zone shape, the diffusing differentiation signal runs into problems with cell population kinetics. Assuming a mechanism to stop the GC reaction in the signaling model may save this concept. Such a mechanism is not difficult to imagine. This could be achieved by a time dependent proliferation rate (Hollowood and Goodlad, 1998), or a recycling probability that is reduced in dependence of the average affinity to the antigen. However, a combination of chemotaxis and a diffusing differentiation signal leads to very reasonable results concerning not only the shape of the dark zone (see Fig. 7) but also the GC cell population kinetics (see Fig. 8) and may be favored. This conclusion will have to be revisited when analyzing a possible role of cell adhesion—a process which has been shown before to induce cell sorting.

### References

Beyer, T., Meyer-Hermann, M. and Soff, G. (2002). “A possible role of chemotaxis in germinal center formation.”, *Int. Immunol.* **14**, 1369–1381.

Brandtzaeg, P. (1996) “The B-cell development in tonsillar lymphoid follicles”, *Acta Otolaryngol. Suppl. (Stockh)* **523**, 55–59.

Brauninger, A., Hansmann, M.L., Strickler, J.G., Dummer, R., Burg, G., Rajewsky, K. and Küppers, R. (1999) “Identification of common germinal-center b-cell precursors in 2 patients with both Hodgkins-disease and non-Hodgkins-lymphoma”, *N. Engl. J. Med.* **340**, 1239–1247.

Camacho, S.A., Koscovilbois, M.H. and Berek, C. (1998) “The Dynamic Structure of the Germinal Center”, *Immunol. Today* **19**, 511–514.

Han, S.H., Zheng, B., dal Porto, J. and Kelsoe, G. (1995a) “*In situ* studies of the primary immune response to (4-Hydroxy-3-Nitrophenyl) Acetyl IV. Affinity-dependent, antigen-driven B-cell apoptosis in germinal centers as a mechanism for maintaining self-tolerance”, *J. Exp. Med.* **182**, 1635–1644.

Han, S.H., Hathcock, K., Zheng, B., Kepler, T.B., Hodes, R. and Kelsoe, G. (1995b) “Cellular interaction in germinal centers: roles of CD40-ligand and B7-1 and B7-2 in established germinal centers”, *J. Immunol.* **155**, 556–567.

Han, S., Zheng, B., Takahashi, Y. and Kelsoe, G. (1997) “Distinctive characteristics of germinal center B cells”, *Immunology* **9**, 255–260.

Hollmann, C. and Gerdes, J. (1999) “Follicular dendritic cells and T-cells—nurses and executioners in the germinal center reaction”, *J. Patho.* **189**, 147–149.

Hollowood, K. and Macartney, J.C. (1992) “Reduced apoptotic cell death in follicular lymphoma”, *J. Pathol.* **163**, 337–342.

Hollowood, K. and Goodlad, J.R. (1998) “Germinal center cell-kinetics”, *J. Patho.* **185**, 229–233.

Hostager, B.S., Catlett, I.M. and Bishop, G.A. (2000) “Recruitment of CD40 and tumor necrosis factor receptor-associated factors 2 and 3 to membrane microdomains during CD40 signaling”, *J. Biol. Chem.* **275**, 15392–15398.

Hur, D.Y., Kim, D.J., Kim, S., Kim, Y.I., Cho, D., Lee, D.S., Hwang, Y., Bae, K., Chang, K.Y. and Lee, W.J. (2000) "Role of follicular dendritic cells in the apoptosis of germinal center B cells", *Immunol. Lett.* **72**, 107–111.

Jacob, J., Kassir, R. and Kelsoe, G. (1991) "*In situ* studies of the primary immune response to (4-hydroxy-3-nitrophenyl)acetyl. I. The architecture and dynamics of responding cell populations", *J. Exp. Med.* **173**, 1165–1175.

Jacob, J., Przylepa, J., Miller, C. and Kelsoe, G. (1993) "*In situ* studies of the primary response to (4-hydroxy-3-nitrophenyl)acetyl. III. The kinetics of V region mutation and selection in germinal center B cells", *J. Exp. Med.* **178**, 1293–1307.

Kelsoe, G. (1996) "The germinal center: a crucible for lymphocyte selection", *Semin. Immunol.* **8**, 179–184.

Kesmir, C. and de Boer, R.J. (1999) "A mathematical model on germinal center kinetics and termination", *J. Immunol.* **163**, 2463–2469.

Koopman, G., Parmentier, H.K., Schuurman, H.J., Newman, W., Meijer, C.J. and Pals, S.T. (1991) "Adhesion of human B cells to follicular dendritic cells involves both the lymphocyte function-associated antigen 1/intercellular adhesion molecule 1 and very late antigen 4/vascular cell adhesion molecule 1 pathways", *J. Exp. Med.* **173**, 1297–1304.

Kroese, F.G., Wubbena, A.S., Seijen, H.G. and Nieuwenhuis, P. (1987) "Germinal centers develop oligoclonally", *Eur. J. Immunol.* **17**, 1069–1072.

Küppers, R. (1999) "Identifying the precursors of Hodgkin and Reed-Sternberg cells in Hodgkins-disease—role of the germinal center in B-cell lymphomagenesis", *J. Acquir. Immune Defic. Syndr. Hum. Retrovirol.* **21**, S74–S79.

Küppers, R., Zhao, M., Hansmann, M.L. and Rajewsky, K. (1993) "Tracing B cell development in human germinal centers by molecular analysis of single cells picked from histological sections", *EMBO J.* **12**, 4955–4967.

Lindhout, E., Koopman, G., Pals, S.T. and de Groot, C. (1997) "Triple check for antigen specificity of B cells during germinal centre reactions", *Immunol. Today* **18**, 573–576.

Liu, Y.J., Joshua, D.E., Williams, G.T., Smith, C.A., Gordon, J. and MacLennan, I.C. (1989) "Mechanism of antigen-driven selection in germinal centres", *Nature* **342**, 929–931.

Liu, Y.J., Zhang, J., Lane, P.J., Chan, E.Y. and MacLennan, I.C.M. (1991) "Sites of specific B cell activation in primary and secondary responses to T cell-dependent and T cell-independent antigens", *Eur. J. Immunol.* **21**, 2951–2962.

Liu, Y.J., Barthelemy, C., de Bouteiller, O. and Banchereau, J. (1994) "The differences in survival and phenotype between centroblasts and centrocytes", *Adv. Exp. Med. Biol.* **355**, 213–218.

Marafioti, T., Hummel, M., Anagnostopoulos, I., Foss, H.D., Huhn, D. and Stein, H. (1999) "Classical Hodgkins-disease and follicular lymphoma originating from the same germinal center B-cell", *J. Clin. Oncol.* **17**, 3804–3809.

McHeyzer-Williams, M.G., McLean, M.J., Labor, P.A. and Nossal, G.V.J. (1993) "Antigen-driven B cell differentiation *in vivo*", *J. Exp. Med.* **178**, 295–307.

Meyer-Hermann, M. (2002a) "A mathematical model for the germinal center morphology and affinity maturation", *J. Theor. Biol.* **216**, 273–300.

Meyer-Hermann, M. (2002b) "Does recycling in germinal centers exist?", *Immunol. Cell Biol.* **80**, 30–35.

Meyer-Hermann, M., Deutsch, A. and Or-Guil, M. (2001) "Recycling probability and dynamical properties of germinal center reactions", *J. Theor. Biol.* **210**, 265–285.

Opera, M. and Perelson, A.S. (1996) "Exploring the mechanism of primary antibody responses to t-cell-dependent antigen", *J. Theor. Biol.* **181**, 215–236.

Oprea, M. and Perelson, A.S. (1997) "Somatic mutation leads to efficient affinity maturation when centrocytes recycle back to centroblasts", *J. Immunol.* **158**, 5155–5162.

Oprea, M., van Nimwegen, E. and Perelson, A.S. (2000) "Dynamics of one-pass germinal center models: implications for affinity maturation", *Bull. Math. Biol.* **62**, 121–153.

Pascual, V., Liu, Y.-J., Magalski, A., de Bouteiller, O., Banchereau, J. and Capra, J.D. (1994) "Analysis of somatic mutation in five B cell subsets of human tonsil", *J. Exp. Med.* **180**, 329–339.

Perelson, A.S. and Oster, G.F. (1979) "Theoretical studies of clonal selection: minimal antibody repertoire size and reliability of self-non-self discrimination", *J. Theor. Biol.* **81**, 645–670.

Rundell, A., Decarlo, R., Hogenesch, H. and Doerschuk, P. (1998) "The humoral immune-response to haemophilus-influenzae type-B—a mathematical-model based on T-zone and germinal center B-cell dynamics", *J. Theor. Biol.* **194**, 341–381.

Tew, J.G., Wu, J., Qin, D., Helm, S., Burton, G.F. and Szakal, A.K. (1997) "Follicular dendritic cells and presentation of antigen and costimulatory signals to B cells", *Immunol. Rev.* **156**, 39.

van Eijk, M., Medema, J.P. and de Groot, C. (2001) "Cellular Fas-associated death domain-like IL-1-converting enzyme-inhibitory protein protects germinal center B cells from apoptosis during germinal center reactions", *J. Immunol.* **166**, 6473–6476.

Wedemayer, G.J., Patten, P.A., Wang, L.H., Schultz, P.G. and Stevens, R.C. (1997) "Structural insights into the evolution of an antibody combining site", *Science* **276**, 1665–1669.

Yasuda, M., Taura, Y., Yokomizo, Y. and Ekino, S. (1998) "A comparative-study of germinal center—fowls and mammals", *Comp. Immunol. Microbiol. Infect. Dis.* **21**, 179–189.

Zelenetz, A.D., Chen, T.T. and Levy, R. (1992) "Clonal expansion in follicular lymphoma occurs subsequent to antigenic selection", *J. Exp. Med.* **176**, 1137–1148.

